# The Oppression of the Medical Practitioner

**Published:** 1889-12-14

**Authors:** W. Moore

**Affiliations:** Stourport


### The Oppression of the Medical Practitioner.

By W. Moore, M.R.C.S., LL.D., Stourport.

I read with extreme interest an article in your issue of November 2, "The Doctor's Pulpit." In my humble opinion it was full of home truths for medical men to digest, and pointed out very ably the great need of combination in the medical profession. Now, at the present day there is one very grave injury by which the general medical practitioner is heavily oppressed, and for which the only possible remedy is a combination practically of the whole of the medical profession throughout the United Kingdom. The evil I refer to is a combination (which is greatly increasing throughout England) of the working and the lower-middle classes to obtain medical and surgical attendance on the cheap—*i.e.*, at a very low rate of payment. These medical aid associations (as they are termed) have amongst their members the skilled artisan earning £2 and upwards per week, well-to-do publicans and other tradespeople, farmers, and numberless other persons, who hitherto have been able and willing to pay reasonable fees for medical attendance. The usual payment by members of these societies is 4s. per annum for an adult and 2s. for a child. For this they are provided with medical and surgical attendance and medicines. A medical officer, usually a stranger to the district, is appointed at a small salary.

One result of such an association in a district is that many private practitioners suffer from much diminished incomes, and others (probably one out of four or five) are completely ruined—*i.e.*, cannot make a living by their work. Another result is that medical men are, in self-defence, frequently obliged to resort to actions altogether lowering to the status of members of a learned profession, such as lessening already small fees (underselling, in fact), starting 1d. and ½d. weekly clubs, touting for patients, etc. A further result is that these associations, by having one medical officer at a small salary doing the work, which (before their formation) provided a fair income to two or more medical practitioners, limit the scope of professional work, and thus render the already very serious question, of what is to become of all the men entering the ranks of the profession, a very much more difficult one to solve.

Undoubtedly, there should exist ample means for members of the working class with small wages and persons in really narrowed circumstances to obtain all necessary medical and surgical attendance at a small rate of payment, and I maintain that the Poor Law system, the existing hospitals, provident clubs, and dispensaries, and such like societies (reformed) are fully sufficient to meet all such wants. In justice to the medical profession, the abuse of the hospital out-patient department should be remedied, and all provident dispensaries, clubs, etc., should have a strictly enforced wage-limiting rule as regards their membership, and all properly qualified medical men practising in the district of a dispensary, etc., should have the option of acting as medical officers of the same.

Before, however, this much to be desired end is reached, there must be practical unanimity amongst medical men. The great question of the present day for the medical profession to solve is how can this cohesion and unanimity be brought about? Combination there must be; the difficulty is how to start the formation of it. Any help or suggestions through your journal would be most gratefully received.

